# Effect of high intratesticular estrogen on global gene expression and testicular cell number in rats

**DOI:** 10.1186/1477-7827-8-72

**Published:** 2010-06-23

**Authors:** Nafisa H Balasinor, Ryan D'Souza, Padma Nanaware, Susan Idicula-Thomas, Neelam Kedia-Mokashi, Zuping He, Martin Dym

**Affiliations:** 1 Neuroendocrinology Division, National Institute for Research in Reproductive Health, Parel, Mumbai, India; 2 Biomedical Informatics Centre of ICMR, National Institute for Research in Reproductive Health, Parel, Mumbai, India; 3 Georgetown University Medical Center, Department of Biochemistry and Molecular & Cellular Biology, 3900 Reservoir Road, NW, Washington, DC 20057, USA

## Abstract

**Background:**

The identification of estrogen receptors alpha and beta and aromatase in the testis has highlighted the important role of estrogens in regulating spermatogenesis. There is a wealth of information on the deleterious effects of fetal and neonatal exposure of estrogens and xenoestrogens in the testis, including spermiation failure and germ cell apoptosis. However, very little is known about gene transcripts affected by exogenous estradiol exposure in the testis. The objective of the present study was to unveil global gene expression profiles and testicular cell number changes in rats after estradiol treatment.

**Methods:**

17beta-estradiol was administered to adult male rats at a dose of 100 micrograms/kg body weight in saline daily for 10 days; male rats receiving only saline were used as controls. Microarray analysis was performed to examine global gene expression profiles with or without estradiol treatment. Real time RT-PCR was conducted to verify the microarray data. In silico promoter and estrogen responsive elements (EREs) analysis was carried out for the differentially expressed genes in response to estradiol. Quantitation of testicular cell number based on ploidy was also performed using flow cytometry in rats with or without estradiol treatment.

**Results:**

We found that 221 genes and expressed sequence tags (ESTs) were differentially expressed in rat testes treated with estradiol compared to the control; the microarray data were confirmed by real time RT-PCR. Gene Ontology analysis revealed that a number of the differentially expressed genes are involved in androgen and xenobiotic metabolism, maintenance of cell cytoskeleton, endocytosis, and germ cell apoptosis. A total of 33 up-regulated genes and 67 down-regulated genes showed the presence of EREs. Flow cytometry showed that estradiol induced a significant decrease in 2n cells (somatic and germ cells) and 4n cells (pachytene spermatocytes) and a marked increase in the number of elongated and elongating spermatids.

**Conclusions:**

This study provides a novel insight into the molecular basis for spermiation failure and apoptosis caused by 17beta-estradiol and it also offers new mechanisms by which adult exposure to environmental estrogens can affect spermatogenesis and fertility.

## Background

Estrogen has often been referred to as a traditionally female hormone. However, there is a growing interest in studying this hormone in males due to the following two main reasons: 1) the discovery of estrogen receptors alpha (ERα), estrogen receptor beta (ERβ), and aromatase in the testis; 2) the influences of estrogen or estrogen like compounds on male fertility. In the adult rat testis, ERα is localized to Leydig cells, whereas ERβ is localized to Sertoli cells and most germ cells. Aromatase is expressed in Leydig cells, Sertoli cells, and germ cells from pachytene spermatocytes to elongated spermatids (reviewed in [1]). In the ERα knockout mice, both sperm morphology and function are affected due to the epididymal hypo-osmolality [2-4]. The inability to absorb fluids in the ERα knockout mice resulted in a generation of backpressure, which affected the seminiferous tubule architecture and function [5]. Loss of fertility in humans was also observed with mutations in ERα [6]. On the other hand, ERβ knockout mice were fertile [7]. The role of estrogens in spermatogenesis was also highlighted by the observations of impaired fertility in aromatase knockout mice. Notably, a progressive decrease in fertility with age was observed in the aromatase knockout mice. Mice deficient in aromatase developed disruptions to spermatogenesis between 4.5 months and 1 year. Spermatogenesis was arrested primarily at early spermiogenic stages, as characterized by an increase in apoptosis and the appearance of multinucleated cells, and a significant reduction in round and elongated spermatids, but no changes were observed in Sertoli cells or earlier germ cells, reflecting the requirement of estrogen for later stages of spermatogenesis [8].

There is growing evidence suggesting a decline in fertility in humans and also an increased incidence of testicular cancer after exposure to environmental estrogen and endocrine disruptors (reviewed in [9]). Studies on boys born from mothers treated with diethylstilbestrol, a very potent estrogen agonist from 1950 to 1970, have reported alterations in sperm quality and higher incidence of genital malformations, cryptorchidism, and testicular cancer compared to the control population [10,11].

Subsequently, in an attempt to decipher the cause of these effects on the testis, several studies were initiated in animal models where they were treated with estrogen or estrogen like compounds in fetal or neonatal life. These estrogenic drugs were administered by injections, gavage, or via drinking water resulting in varied effects, such as a decrease in Sertoli cell number, Leydig cell hyperplasia, a decreased sperm count, and a decrease in testicular weight [12,13]. Most of these studies focused on the administration of estrogens/xenoestrogens during fetal or neonatal life, and subsequent effects in adult animals were observed. Gill-Sharma et al. observed that 17β-estradiol treatment at a dose of 100-1,000 μg/kg/day to adult male rats for 60 days resulted in complete azoospermia [14]. Similarly, Toyama et al. showed that administration of estradiol benzoate at a dose of 10-160 μg/kg/day for different periods (2 days - 8 weeks) resulted in a loss of spermatids beyond step 6 with an effect on the ectoplasmic specializations [15]. In an attempt to study the possible effects of estrogen on spermatogenesis in adult rats, we administered 17β-estradiol to adult male rats for ten days at a dose of 100 μg/kg/day. This dose has been previously shown to cause a four-fold increase in intratesticular estrogen levels and a concomitant suppression of intratesticular testosterone to below 10% of control values; circulating follicle stimulating hormone (FSH) fell to below 50% of control values [16,17]. Principally, we observed a stage specific effect of treatment where germ cells in stages I-V of the cycle of the seminiferous epithelium remained unaffected [16,17]. However, in stages VII-VIII of the cycle, spermatids were seen in deep recesses of the epithelium and those that moved to the lumen failed to be released [16,17]. Failure of spermiation was attributed to the absence of tubulobulbar complexes, a testis specific cell junction involved in sperm release [16,17]. In addition, an effect on the Sertoli cell cytoskeleton was observed. A significant increase in TUNEL positive germ cells in stages VII-XIV of the cycle was also observed [16,17].

To our knowledge there are no studies showing the effects of estrogen on changes in global gene expression in adult mammalian spermatogenesis in vivo. Given the fact that environmental estrogens could affect the process of germ cell maturation either by affecting Sertoli cell function or germ cells directly, the objective of this study was to identify changes in gene expression and testicular cell number directly affected by high intratesticular estrogens. This study could provide a novel insight into the molecular basis for spermiation failure and germ cell apoptosis caused by estradiol.

## Methods

### Animals

Holtzman male rats (75-day-old) were maintained at a temperature of 22-23°C, humidity at 50%-55%, and a light/dark cycle of 14 h:10 h. Food and water were available *ad libitum*. The animals were maintained in the animal house of the institute (National Institute for Research in Reproductive Health, Parel, Mumbai, India). The use of animals in this study was approved by the Institute's Ethics Committee for Care and Use of Laboratory Animals for Biomedical Research.

### Experimental design and steroid treatment

17β-estradiol (Sigma, St. Louis, MO) was administered to 10 adult male rats at a dose of 100 μg/kg body weight daily for 10 days. The drug was suspended in saline and administered subcutaneously as described previously [17]. Ten male animals receiving only saline were used as controls. For flow cytometric analysis, two doses of 17β-estradiol treatment were used, i.e., 20 and 100 μg/kg body weight for 10 days, in 10 male rats in each dose group, while another 10 animals with only saline were used as controls.

### Testis tissue collection

The control and 17β-estradiol-treated animals were grouped into three batches (each batch with 3 or 4 rats). Batch 1: For microarray studies, 4 animals were decapitated, testis were excised and placed in RNA LATER (Ambion, USA). Batch 2: For real time RT-PCR studies, 3 animals were decapitated; testes were excised and frozen in liquid nitrogen until use. Batch 3: For flow cytometry analysis, 3 animals were decapitated, testis excised and used for the preparation of single cells as described below.

### Microarray analysis

Total RNA was extracted from the testes of 17β-estradiol-treated and -untreated rats utilizing the Trizol reagent (Invitrogen, San Diego, CA) pursuant to the manufacturer's protocol and purified using the RNeasy Mini kit (QIAGEN Inc., Valencia, CA). The concentration and purity of total RNA and biotin-labeled cRNA were evaluated by measuring the 260/280 nm ratios, and the integrity of total RNA and the quality of biotin-labeled cRNA were assessed by 1.0% denaturing agarose gel electrophoresis. Total RNA with a 260/280 nm ratio of approximately 2.0 was used to generate biotin-labeled cRNA target for the Rat Genome U34A Array (Affymetrix, Santa Clara, CA). Seven micrograms of total RNA were reverse-transcribed into single strand cDNA that was subsequently converted to double strand cDNA and then purified by the use of the GeneChip Sample Cleanup Module kit (Affymetrix). The purified double strand cDNA served as a template to synthesize biotin-labeled cRNA using the BioArray High Yield RNA Transcription Labeling kit (Enzo Life Sciences, Inc., Farmingdale, NY, USA), and the biotin-labeled cRNA was purified using the GeneChip Sample Cleanup Module kit (Affymetrix) and then fragmented to 35-200 base fragments according to the Affymetrix protocol. The Rat Genome U34A Array containing 8,799 gene transcripts was loaded with 200 μl of the hybridization cocktail containing 15 μg of the fragmented biotin-labeled cRNA and then hybridized for 16 h at 45°C. After hybridization, the arrays were washed, stained with streptavidin phycoerythrin using the Affymetrix Genechip Fluidics Workstation 400, and scanned on a Hewlett-Packard Gene Array scanner (Hewlett-Packard Co., Palo Alto, CA). Finally, raw data were generated and analyzed using the GeneSpring 5.0 software (Silicon Genetics, CA). Genes with a fold change of 2 or more were considered as differentially expressed and Gene Ontology grouping was performed according to their molecular function using the GeneSpring 5.0 software.

### Real time RT-PCR

Testicular total RNA was extracted from the 100 μg/kg estradiol-treated rat testis and the control using Trizol reagent. The RNA was treated with DNase to remove potential DNA contamination and cDNA was synthesized using Reverse Transcriptase system and oligo DT primers (Promega, USA). For real time PCR, 1 μl of cDNA was mixed with SYBR green Master Mix (Bio-Rad, USA) in 25 μl of reaction mixture. Primers were designed using Primer 3 software described previously [16,17]. The primer sequences for the chosen genes are listed in Additional File 1, Table S1. Standard curves were prepared from serially diluted cDNA to check the efficiency of the reaction. Housekeeping gene L19 was used as an internal control. Since the efficiency was similar for all genes with the housekeeping gene, the fold change was determined using the modified 2^-ΔΔCT ^method, i.e., ΔC_T _method using a reference gene as indicated in the Bio-Rad laboratories application guide. The formula was as follows. Ratio (reference/target) = 2^C^_T_^(reference) - C^_T_^(Target) ^, where C_T _(reference) = C_T _values of house keeping gene L19, and C_T _(Target) = C_T _values of target gene. Relative expression of each gene in the control was designated as 100, and each sample was run in triplicates. Melt curve analysis was carried out to check for the presence of a single PCR product, which was also confirmed by electrophoresis on a 2% agarose gel (data not shown).

**Table 1:** Gene ontology grouping of the differentially expressed genes in response to 17β-estradiol

Molecular Function	Gene Name	Fold Change	*Localization in the Testis	Function	Ref.
**I) Metabolism (androgen and xenobiotic)**	1) Steroidogenic acute regulatory protein *(Star)*	-5.45	Leydig cells, Sertoli cells	Carrier protein involved in transport of cholesterol to the inner mitochondrial membrane	[53]
	2) Hydroxysteroid 11-beta dehydrogenase-1*(Hsd11b1)*	-2.10	Interstitial cells	Androgen & estrogen metabolism	[54]
	3) Alcohol dehydrogenase-1 *(Adh1)*	-3.28	Leydig and Sertoli cells	Enzyme catalyzing the rate limiting step in the conversion of retinol to retinoic acid	[28]
	4) Carboxylesterase-3 *(Ces3)*	-26.85	Leydig cells	Synthesis of testosterone; protects Leydig cells from damage by toxins	[26]
	5) Cytochrome P450, family-1, subfamily-b, polypeptide-1 *(Cyp1b1)*	-2.41	Leydig cells	Involved in xenobiotic metabolism	[27]
	6) Sulfotransferase family 1A, phenol-preferring, member-1 *(Sult1a1)*	-2.12	Leydig cells	Involved in xenobiotic metabolism	[55]
**II) Cytoskeletal maintenance**	1) Actin related protein 2/3 complex, subunit-1B *(Arc 1B or p41Arc)* Actin related protein 2/3 complex, subunit-5-like (predicted) *(Arpc5L)*	-2.46 -2.01	Sertoli cells	Part of the Arp2/3 complex; contributes to the stability of the complex and are involved in the *de novo *polymerization of actin	[31,32]
	2) ENA Vasodilator Phosphoprotein *(Evl) *	-2.12	Germ cells*	Actin binding protein involved in actin dynamics	[56]
	3) Capping protein gelsolin like *(Capg)*	-2.42	Ectoplasmic specialization	An actin severing protein	[33]
	4) Tubulin beta-5 *(tubb5)*	-2.22	Sertoli and germ cells*	Component of microtubules which is responsible for spermatid translocation in the mammalian seminiferous epithelium	[57]
	5) dynein cytoplasmic light intermediate polypeptide *(Dncli2)*	-2.29	Sertoli and germ cells*	Component of dynein, the motor protein, responsible for spermatid translocation in the mammalian seminiferous epithelium and Sertoli cell protein transport	[57]
	6) casein kinase-2 beta subunit *(Csnk2b)*	-2.34	Sertoli and germ cells*	Disruption of *Csnk2a *results in infertility, globozoospermia and retention of defective spermatids	[35]
**III) Intracellular transport and endocytosis**	1) Syntaxin-5a *(Stx5a)*	-2.22	Sertoli cells*	Involved in Golgi function, cytokinesis, and spermatid differentiation in drosophila	[58]
	2) Syntaxin -8 *(Stx8)*	-2.10	Spermato-gonia and Sertoli cells*	Involved in vesicular transport and membrane fusion events necessary for protein transport from early endosomes to late endosomes.	[59]
	3) Adaptor-related protein complex-2, sigma-1- subunit *(Ap2S1)*	-2.07	Sertoli and germ cells*	*Ap2S1 *gene codes for the sigma subunit of the AP2 complex and helps in targeting of the complex to membranes. AP2 complex is known to trigger the formation of the clathrin lattice machinery at the plasma membrane	[37]
	4) Ral A binding protein *(Ralbp1)*	-2.06	Spermato-gonia & Sertoli cells*	RalBP1 is a putative effector protein of Ral and possesses the GTPase-activating activity for Rac1 and CDC42 is a critical component of clathrin-coated pit-mediated endocytosis	[60]
	5) trafficking protein particle complex 1 (predicted) *(Trappc1) *	-2.01	Sertoli and germ cells*	Multisubunit tethering complex involved in vesicle mediated transport	[61]
	6) Lysosomal membrane glycoprotein-2 *(Lamp 2)*	-2.00	Tubulobulbar complexes	Lamp 1 and 2 are involved in lysosome biogenesis, autophagy, and cholesterol homeostasis. Lamp 1 is speculated to be involved in endocytosis and internalization of junctions	[39,40]
	7) Phosphatidylinositol binding clathrin assembly protein *(PiCalm)*	+2.41	Germ cells and very little expression in Sertoli cells*	Homologue of the AP180 protein, it plays a significant role in the clathrin internalization machinery and over expression of the protein destabilizes the clathrin complex thus disrupting clathrin mediated endocytosis	[38]
**IV) Iron metabolism**	1) Haptoglobin (*Hp*)	9.25	Leydig cells, Sertoli cells and germ cells	Hp protein is speculated to be involved in the recycling of heme groups hence involved in the maintenance of Sertoli cell function	[43]
	2) Hemoglobin alpha adult chain-1 (*Hba-a1*) and beta chain complex (*Hbb*).	-8.85 -9.89	Spermato-gonia*	Possible influence on germ cell number	[44]
	3) Solute carrier family-39 (iron-regulated transporter), member-1 (Slc40a1)	+2.21	Round spermatids and Sertoli cells*	Basolateral iron transporter family	[45]
**(V) Germ cell apoptosis**	1) Nitric oxide synthase-3, endothelial cell *(Nos3)*	+4.02	Degenerating germ cells	Calcium dependant enzymes which probably has a role in apoptosis	[47,48]
	2) Retinoblastoma- 1 *(Rb1)*	+2.73	Sertoli cells and spermato-gonia*	Regulates G1/S transition in cell cycle	[62]
	3)Transforming growth factor beta receptor-3 *(Tgfbr3)*	+2.17	All testicular cell types	Transmission of extracellular TGF signal into the cell and induce apoptosis in gonocytes of fetal testis *in vitro *to control germ cell numbers during fetal life	[49,50]
	4) Peroxiredoxin-3 and -6 *(Prdx3 and Prdx6)*	-2.81 -2.73	Spermato-gonia and spermato-cytes with maximal expression in pachytene spermato-cytes	Oxidative stress related proteins which are cytoprotective and know to maintain mitochondrial integrity; decrease in Prdx3 is known to sensitize cells to apoptotic stimulus	[52,63]

'*' Testicular localization of most of the genes listed in the table was obtained from the Mammalian Reproductive Genetics database [44].

### In-silico promoter and estrogen responsive elements (ERE) analysis

In silico promoter and ERE analysis was carried out for the differentially expressed genes. Initially, an *in house *Perl code was used to retrieve the sequence information of the differentially expressed genes in the FASTA format. These sequences were then analyzed for the presence of ERE binding sites based on the information present in the TRANSFAC database [18] using the Dragon ERE Finder version 2 [19] and MATCH algorithm [20]. Genomatix Software GmbH (Germany) was also used. The promoters regions were identified by submitting sequences 3,000 bp upstream and 500 bp downstream from the transcription start sites (TSS sites). The identified promoters were subjected to ERE analysis. An ERE is defined as a site which contains the 17 bp consensus sequence [2n GGTCA 3n TGACC2n where n stands for any nucleotide] [21]. It should be noted that the estrogen receptors can also bind to half ERE sites.

### Flow cytometric analysis

Since microarray analysis revealed that a number of genes were involved in cell apoptosis, differential testicular cell number was performed based on ploidy using flow cytometry, according to the protocol described previously [22]. Two doses of 17β-estradiol treatment were used, i.e., 20 and 100 μg/kg body weight for 10 days. A cell mixture containing male germ cells and somatic cells was obtained from 17β-estradiol treated rats and controls pursuant to the procedure described previously [23,24]. Cells were washed twice with PBS and treated with 1 ml of staining solution containing 0.1% NP 40 solution in PBS containing 25 μg/ml of propidium iodide (Roche, Germany) and 40 μg/ml of RNase (Sigma) for 20 min in the dark at 37°C. The cells were then filtered with a 40 μm mesh and run on a FACS Vantage SE (BD Biosciences) equipped with an argon laser. As an internal control, thymus cells were used to set the instrument. Ten thousand cells were counted for each sample.

### Statistical analysis

For microarray analysis, the raw data were normalized and filtered to exclude the absent genes. Only the present or marginal genes in both 17β-estradiol-treated rats and the control were used to compare their differential expression. The analysis of variance (ANOVA) and the 't' test as included in the GeneSpring 5.0 software were employed to determine the differentially expressed genes between 17β-estradiol-treated groups and the control, and p < 0.05 was set to show statistically significant differences. All values for real time RT-PCR and flow cytometry were presented as mean ± SEM, and statistically significant differences (p < 0.05) between 17β-estradiol-treated groups and the control were determined by the ANOVA and the students unpaired 't' test using Graph pad prism (GraphPad Inc., San Diego, CA).

## Results

### Analysis of the differentially expressed genes by microarray

**Figure 1:**
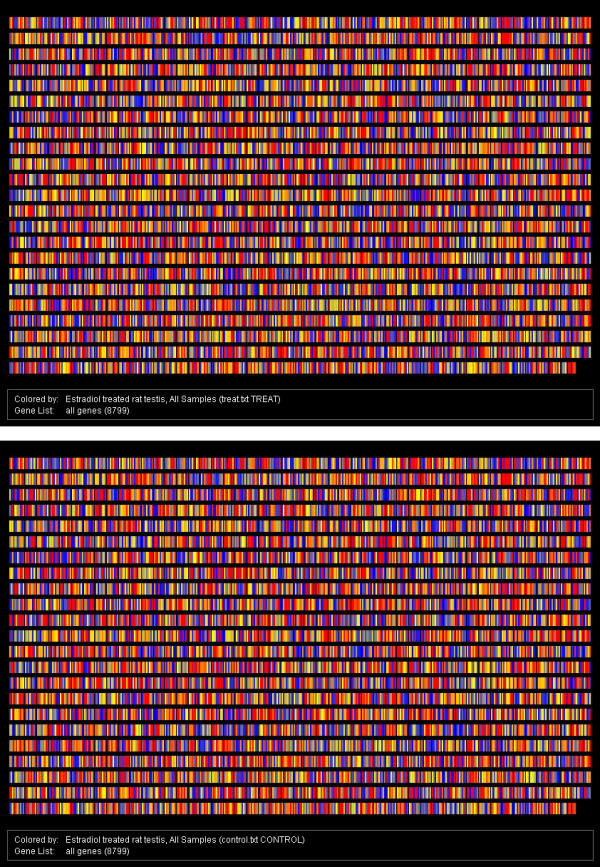
**Global gene expression profiles in rat testis with 17β-estradiol treatment (upper panel) compared to the control (lower panel) using microarray analysis**. Each spot represents a specific probe set of 8,799 genes at the Rat Genome U34A Array. Spots in blue indicate weak levels of gene expression, and spots in red denote high levels of gene expression. Spots in yellow indicate middle levels of gene expression.

We first examined the gene expression patterns in rat testis with 17β-estradiol treatment compared to the control using microarray analysis (Figure 1). Out of a total of 8,799 genes present on the Rat Genome U34A Array, 3,659 genes and expressed sequence tags (ESTs) were expressed in the control, and 3,613 genes and ESTs were expressed in 17β-estradiol treated rat testes. Among them, 221 genes and ESTs were differentially expressed with 2 or more fold changes in rat testis with 17β-estradiol treatment compared to the control. In total, we found that 75 annotated genes and ESTs were up-regulated by 17β-estradiol (See Additional File 2, Table S2) and 146 annotated genes and ESTs were down-regulated by 17β-estradiol (See Additional File 3, Table S3).

**Table 2:** ERE analysis of promoters of the differentially expressed genes by 17β-estradiol

Gene symbol	Gene ID	Core sim.	Matrix sim.	Sequence
Star	25557	1	0.889	aggaaccAAGGccagccag
Star	25557	1	0.92	tgagttcAAGGccagcctg
Hsd11b1	25116	1	0.875	ttcttgcAAGGccattgct
Hsd11b1	25116	1	0.893	cagagacAAGGccagagag
Adh1	24172	0.808	0.831	ctggggcagctcCACCttt
Cyp1b1	25426	1	0.843	ctggGTCAccctgagcaca
Cyp1b1	25426	1	0.894	gcagGTCAgtctgtccacg
Cyp1b1	25426	1	0.895	cgtggacagactGACCtgc
Cyp1b1	25426	1	0.902	gttcttgAAGGtcaggggt
Sult1a1	83783	1	0.947	caggagcAAGGtcagggaa
Sult1a1	83783	0.767	0.85	acagGACAaggtggccacc
Sult1a1	83783	0.794	0.837	ggtggccaccttGTCCtgt
Arpc1b	54227	1	0.878	gtagGTCAtagtcccttgt
Evl	79115	1	0.871	tggaacaAAGGtcaaaggc
Capg	297339	1	0.832	agctGTCAcattgccctga
Capg	297339	1	0.836	tcagGTCAtgcttagcttc
Stx5a	65134	1	0.909	cgagcccAAGGccacccgc
Ralbp1	84014	1	0.891	gaggtccAAGGgcatccat
Ralbp1	84014	1	0.883	tcttcagAAGGtcactgtg
Ralbp1	84014	1	0.872	agcagtaAAGGacacgaaa
Picalm	89816	1	0.889	AgtatgcAAGGccacgacc
Hp	24464	1	0.887	ccttgctAAGGtcagtgac
Hp	24464	1	0.839	tcggGTCAtggtgctccct
Hp	24464	1	0.826	agggagcaccatGACCcga
Hp	24464	1	0.887	ccttgctAAGGtcagtgac
Hbb	24440	0.808	0.847	tggggtcaacaaAACCtcc
Nos3	24600	0.808	0.836	CagggccaagccCACCcca
Prdx3	64371	1	0.9	gctgGTCAgagtgtctgtt
Prdx3	64371	1	0.825	aacagacactctGACCagc

**Figure 2:**
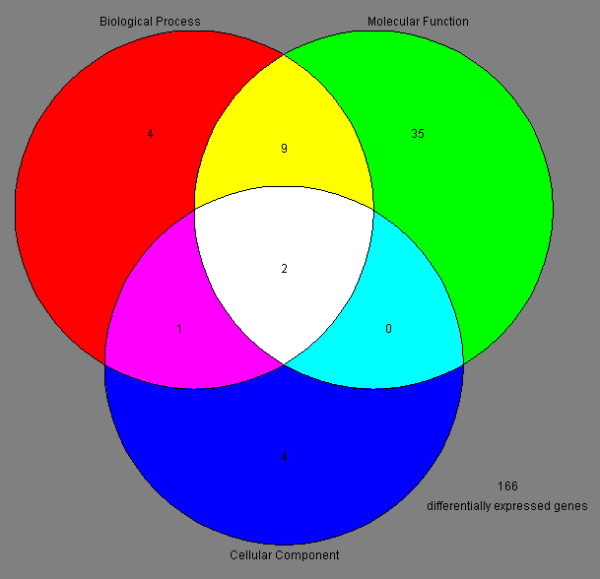
**Gene ontology grouping analysis of 221 differentially expressed genes in response to 17β-estradiol**. Venn diagram showed three major functions of the differentially expressed genes induced or repressed by 17β-estradiol.

Differentially expressed genes by 17β-estradiol were then grouped on the basis of molecular function using the Gene Ontology program in the GeneSpring 5.0 software. Gene ontology grouping analysis revealed that 55 genes have known functions: a) 16 genes are involved in biological process; b) 46 genes were related to molecular function; c) 7 genes are involved in cellular component (Figure 2). Additionally, 166 genes and ESTs have unknown functions (Figure 2). Interestingly, a number of the differentially expressed genes were found to be involved in androgen and xenobiotic metabolism, cytoskeletal maintenance, intracellular transport and endocytosis, iron binding and transport, and germ cell apoptosis (Table 1). For example, 6 gene transcripts, including *Star*, *Hsd11b1*, *Adh1*, *Ces3*, *Cyp1b1*, and *Sult1a1 *were down-regulated by estradiol, and these genes are present in the somatic cells of testis and they are involved in androgen and xenobiotic metabolism (Table 1). We also found that germ cell apoptosis genes, including *Nos3*, *Rb1*, *Tgfbr3*, were up-regulated by estradiol and they are expressed in both germ cells and somatic cells, whereas *Prdx3 *and *Prdx6 *gene transcripts were down-regulated by estradiol and they are present only in germ cells (Table 1). These results suggest that estradiol regulates the transcription of numerous genes that play potential roles in regulating somatic cell and germ cell fate determinations including germ cell apoptosis.

### Validation of microarray data by real-time RT-PCR

**Figure 3:**
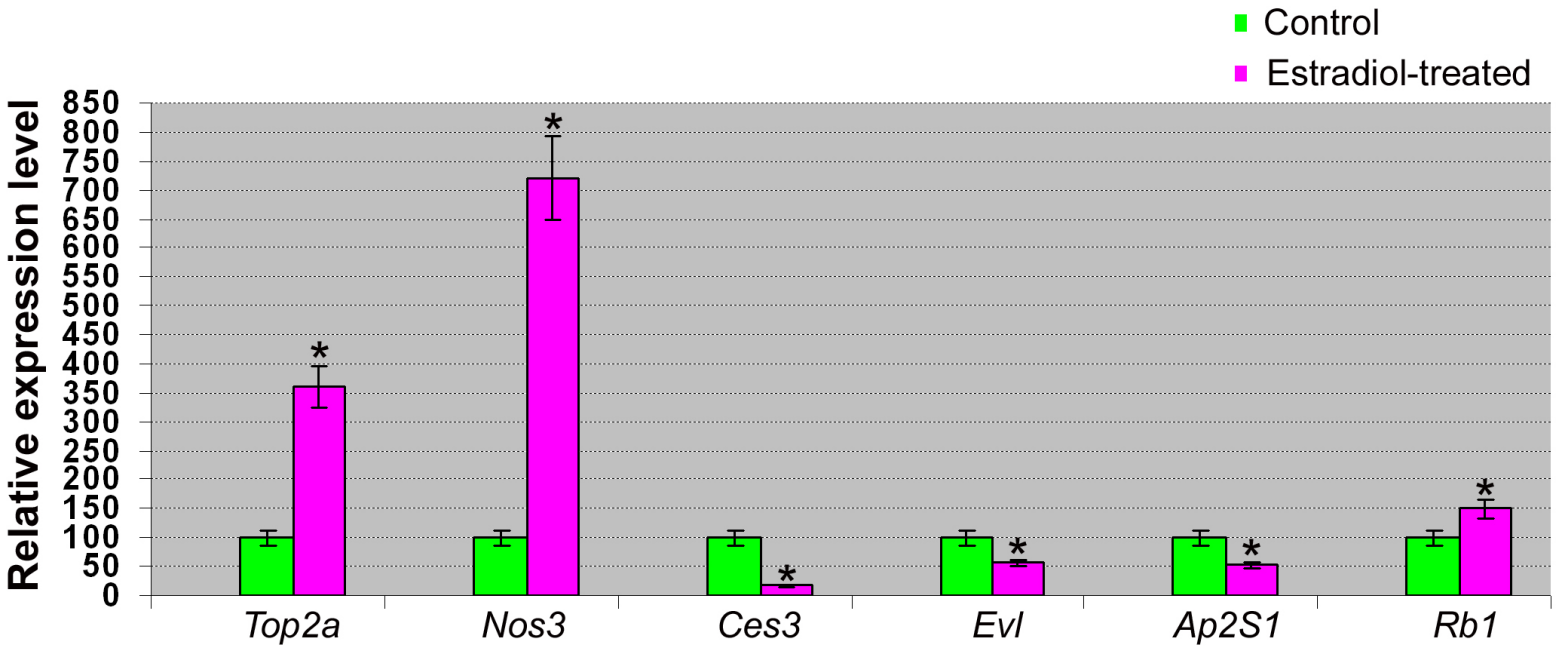
**Validation of microarray results using real time RT-PCR**. Six genes were randomly selected. Data are represented as expression fold change calculated as a ratio (reference/target) as detailed in the Materials & Methods. Relative expression of each gene in the control was designated as 100, and values are mean ± SEM. '*' represents a statistically significant difference (p < 0.05) between 17β-estradiol treated rat testis and the control.

To confirm the microarray data, we chose randomly six differentially expressed genes (estradiol up- and down-regulated genes), namely topoisomerase (DNA) 2 alpha (*Top2a*), nitric oxide synthase 3, endothelial cell or eNOS (*Nos3*), carboxyesterase3 (*Ces3*), ENA Vasodilator Phosphoprotein (*Evl*), adaptor-related protein complex 2, sigma 1 subunit (*Ap2S1*), and retinoblastoma (*Rb1*). As shown in Figure 3, real time RT-PCR results were in agreement with the microarray data. The trend of expression in the real time RT-PCR data was similar to the microarray data. The fold changes generated by microarray and real time RT-PCR did not necessarily match due to these two different approaches used.

### ERE analysis on promoters of the differentially expressed genes in response to 17β-estradiol

In an attempt to identify genes directly regulated by estrogen, promoters of the differentially expressed genes in response to 17β-estradiol were scanned. The promoter scan analysis occasionally gave multiple promoter sequences for particular genes due to the presence of splice variants. Each of these promoters was scanned for the presence of EREs. A total of 33 up-regulated genes and 67 down-regulated genes showed the presence of EREs. The sequences with a high matrix and core similarity represent those with the highest conservation of bases of the candidate sequence to the consensus sequence. The EREs of genes listed in Table 1 are provided in Table 2. Estradiol-up and -down-regulated genes showed the presence of EREs in their promoters (See Additional File 4, Table S4 and Additional File 5, Table S5, respectively).

### Effect of estrogen on male germ cell and somatic cell numbers in the testis by flow cytometry

**Figure 4:**
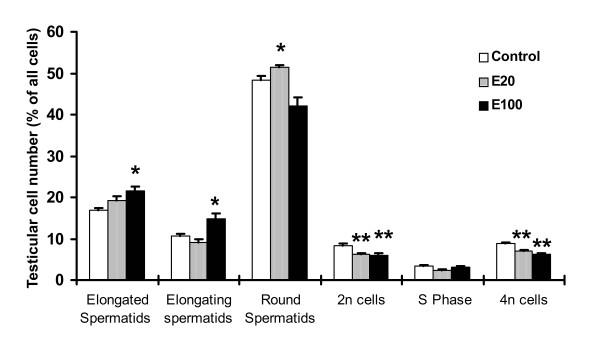
**Graphical representation of testicular cell numbers after 17β-estradiol treatment**. 2n represents somatic cells and some germ cells, 4n represents pachytene spermatocytes, and S represents the cells in the DNA synthetic phase of the cell cycle. E20 and E100 represent adult male rats treated with a dose of 20 and 100 μg/kg/day of 17β-estradiol for ten days, respectively. '*' denotes an increase of statistically significant differences (p < 0.05) with respect to the control, and '**' denotes a decrease of statistically significant differences (p < 0.05) with respect to the control.

As shown in Figure 4, we observed that treatment with 17β-estradiol resulted in a significant decrease in 2n cells composed of somatic and male germ cells and 4n cells composed of pachytene spermatocytes in both dose treatment groups compared to the controls. In direct contrast, there was a significant increase in number of elongated and elongating spermatids compared to the controls, which reflects the retention of mature spermatids that fail to be released from the lumen.

## Discussion

The identification of aromatase and estrogen receptors on testicular germ cells has aroused great interest in the traditionally female hormone in spermatogenesis (reviewed in [1]). Our previous studies revealed that the administration of exogenous 100 μg/kg/day 17β-estradiol for 10 days in rats resulted in a high level of intratesticular estrogen [17]. An increase in germ cell apoptosis and the presence of spermatids in the deep recess at stages VII-VIII culminating in failure of spermiation were observed [16,17], which is directly due to a high level of intratesticular estrogen in rats induced by exogenous 17β-estradiol for 10 days. The present study was designed to identify global gene transcripts affected by a high level of intratesticular estrogen so as to elucidate the molecular basis of the early observed effects. Gene ontology grouping analysis of the differentially expressed genes revealed that a number of genes involved in metabolism, iron binding and transport, cytoskeletal maintenance, intracellular transport, endocytosis, and cell apoptosis, were affected by 17β-estradiol. Specifically, we found that a number of genes involved in androgen metabolism were down-regulated. These genes include steroidogenic acute regulatory protein (*Star*), hydroxysteroid 11-beta dehydrogenase 1 (*Hsd11b1*), and alcohol dehydrogenase 1 (*Adh1*). The decrease in the expression of these genes could lead to the decrease in androgen synthesis and thus results in the earlier observed decrease in intratesticular and serum testosterone. Strauss et al. [25] have reported similar observations in aromatase over-expressing mice.

17β-estradiol treatments also significantly decreased the expression of carboxylesterase 3 (*Ces3*), which is related to testosterone synthesis and the protection of Leydig cells from damage by toxins [26]. Two other genes involved in xenobiotic metabolism were down regulated, namely cytochrome P450, family 1, subfamily b, polypeptide 1 (*Cyp1b1*) and sulfotransferase family 1A, phenol-preferring, member 1 (*Sult1a1*). Recently *Cyp1b1 *was shown to be abundantly expressed in the testis specifically in Leydig cells [27]. ERE analysis on promoter sequences revealed the presence of EREs for *Star, Hspd11b1, Acat 2, Cyp1b1*, and *Sult1a1*, suggesting a direct effect of estrogens on these genes.

Beside androgen metabolism, type 1 alcohol dehydrogenase enzymes (*Adh1*) is also involved in the conversion of retinol to retinoic acid, and in situ hybridization studies have revealed that the mRNA for this gene is selectively expressed in Sertoli and Leydig cells [28]. Spermiation failure has been observed in rats fed on Vitamin A deficient diet [29]. Interestingly in our array data, *Adh1 *was down-regulated, suggesting its possible influence on spermiation.

Several genes required to maintain cytoskeletal integrity were down-regulated by estradiol treatment. Our earlier study demonstrated that the process of spermiation was affected due to an influence on the cytoskeletal network [16]. These genes can be grouped into two categories based on their function in affecting microfilament or microtubules. The first category represents those whose protein products affect microfilament stability and include genes such as actin related protein 2/3 complex, subunit 1B (*Arc 1B*), actin related protein 2/3 complex, subunit 5-like (predicted) (*Arpc5L*), ENA Vasodilator Phosphoprotein (*Evl*), and capping protein gelsolin like (*Capg*). The Arc 1B and Arpc 5 proteins form a part of the Arp2/3 complex which is involved in the de novo polymerization of actin [30]. Both the Arc1B and Arpc 5 play a crucial role in contributing to the stability of the Arp2/3 complex [31]. Vaid et al. [32] have reported that the Arp2/3 complex plays an essential role in the reorganization of actin during tubulobulbar complex (TBC) formation. The down regulation of two crucial proteins of the Arp2/3 complex could result in the destabilization of this complex and improper targeting of this complex to sites of TBC formation. This would eventually affect the formation of TBC during spermiation and lead to failure of spermiation as observed in our previous study [16]. In addition, gelsolin, an actin severing protein with a role in actin filament-containing adhesion complexes known as ectoplasmic specialization [33] and in podosome (structures similar to TBC) formation along with Arp3 in endothelial cells [34], is down-regulated in the present study. The second category includes genes that contribute to microtubule stability or associate with microtubule networks, namely tubulin beta 5 (*tubb5*), dynein cytoplasmic light intermediate polypeptide (*Dncli2*), and casein kinase 2 beta subunit (*Csnk2b*). Casein kinase 2 is an oligomeric protein that has two alpha and two beta subunits and targeted disruption of its alpha subunits results in globozoospermia and retention of defective spermatids [35]. In other cellular systems, the alpha subunit of Casein kinase II is known to bind to microtubules and tubulin heterodimers, exerting a potent effect on microtubule assembly and bundling [36]. Down-regulation of genes coding for the casein kinase beta subunit can be seen as a contributing factor that could lead to our earlier observed disruption in microtubule bundling in the Sertoli cells [16]. Among the genes grouped under this category, *Arc1B*, *Evl*, and *Capg *have an ERE sequence on their promoters, suggesting estrogen regulated genes although their function remains to be determined in the testis.

Several other genes involved in intracellular transport and endocytosis were differentially regulated by estradiol. The majority of these genes, namely syntaxin 5a (*Stx5a*), syntaxin 8 (*Stx8*), *Ap2S1*, Ral A binding protein (*Ralbp1*), trafficking protein particle complex 1 (predicted) (*Trappc1*), and lysosomal membrane glycoprotein 2 (*Lamp 2*), were down-regulated by estradiol. The only up-regulated gene in this group was phosphatidylinositol binding clathrin assembly protein (*PiCalm*). Among these genes, *Ap2s1 *and *PiCalm *are known to play a significant role in cell mediated endocytosis. *Ap2s1 *encodes the sigma subunit of the Adaptor Protein 2 (AP2) complex and helps in targeting the AP2 complex to membranes. The AP2 complex is known to trigger the start of the formation of the clathrin lattice machinery at the plasma membrane [37]. *PiCalm *is a non-neuronal homologue of the AP180 protein and may play a significant role in the clathrin internalization machinery [38]. Lamp proteins are transmembrane proteins that form important components of the lysosomal membrane. These proteins play an essential role in lysosome biogenesis, autophagy, and cholesterol homeostasis. There are two isoforms of Lamp proteins called Lamp 1 and 2. Based on double knock out studies for both Lamp 1 and Lamp 2, it has been proposed that these proteins have partially overlapping functions [39]. In the testis, only the Lamp 1 protein is localized to the TBC, which might be involved in endocytosis and internalization of junctions [40]. Tubulobulbar complexes are also involved in elimination of excess spermatid cytoplasm and recycling of junctions by endocytosis [40]. In our previous study, we observed absence of endocytic vesicles and retention of junctional molecules such as α6β1 integrin along with the failed spermatids in the estradiol-treated group [16], suggesting an effect on endocytosis. ERE elements have been identified on the promoters of the following genes, including *Stx5a, Ralbp1*, and *PiCalm*. The present study suggests that high levels of intratesticular estrogen could inhibit the formation of clathrin-coated pits that is the initial event in formation of the tubulobulbar complexes leading to the observed absence of TBC.

Iron is required by all organisms and male fertility is affected by disruptions in iron balance [41]. In the testis, the transport and delivery of iron to the germ cells from Sertoli cells is mediated by transferrin [42]. In the present study, no change in expression of transferrin was seen; however, several iron binding protein, namely haptoglobin (*Hp*), hemoglobin alpha adult chain 1 (*Hba-a1*), and hemoglobin beta chain complex (*Hbb*), were significantly down-regulated. Hp protein has been identified in the testis specifically in Leydig cells, Sertoli cells, and germ cells, and it is speculated to be involved in the recycling of heme groups, suggesting that it is involved in the maintenance of Sertoli cell function rather than directly in the process of spermatogenesis [43]. Both Hba-a1 and Hbb have been identified to be significantly expressed in spermatogonial cells [44], reflecting their possible influence on maintaining spermatogonial number. Another gene of significance that was up-regulated was the solute carrier family 39 (iron-regulated transporter), member 1 (*Slc40a1*). This protein belongs to the basolateral iron transporter family [45] and its up-regulation can be seen as a means to salvage loss of iron due to the down regulation of other iron binding proteins. It is interesting to note that *Slc40a1 *was down-regulated when FSH was administered to *hpg *mice [46] suggesting that this gene could be negatively regulated by FSH. EREs were identified in the *Hp *and *Hbb *genes promoters. Iron was also reported to be involved in spermiation. Deficiencies in testicular transferrin resulted in reduced levels of spermiation and a decrease in the number of epididymal spermatozoa (Reviewed in [42]). Since we have previously observed spermiation failure with estradiol treatment, it is tempting to speculate an indirect influence of high levels of intratesticular estrogen on factors promoting failure of spermiation.

Of note, several genes that eventually drive germ cells to undergo apoptosis were differentially regulated after estradiol treatment. Among them, *Nos3*, *Rb1*, and *Tgfbr3 *were up-regulated. On the other hand, Peroxiredoxin 3 and 6 (*Prdx3 and Prdx6*) were down-regulated. *Nos3 *has been shown to be significantly expressed in degenerating germ cells in human testis [47]. When transgenic mice overexpressing *Nos3 *and wild type mice were both subjected to unilateral cryptorchidism, the number of spermatocytes and spermatids undergoing apoptosis in the transgenic mice was much higher than the wild type cryptorchid mice, suggesting a role for *Nos3 *in apoptosis [48]. *Tgfbr3 *is known to be expressed in all testicular cell types [49]. Olaso et al. [50] suggested that TGF induces apoptosis in gonocytes of the fetal testis as a means to control germ cell numbers during fetal life. The *Rb1 *gene is known to be expressed in Sertoli cells and spermatogonia in all stages of spermatogenesis, however maximum levels are in stages VII and VIII of the cycle of the seminiferous epithelium. Studies using methoxyacetic acid to induce apoptosis have shown an increase of retinoblastoma protein [51]. Peroxiredoxin are oxidative stress related proteins that are cytoprotective and maintain mitochondrial integrity. A significant decrease in peroxiredoxin 3 sensitizes cells to apoptotic stimulus. Localization studies have shown the presence of peroxiredoxin 3 in spermatogonia and spermatocytes with maximal expression in pachytene spermatocytes [52]. EREs were identified on the promoters for *Prdrx3 *and *Nos3 *genes, suggesting a direct effect of estrogen on apoptosis. Collectively, up-regulation of genes (*Nos3*, *Rb1*, *Tgfbr3*) involved in germ cell apoptosis, together with down-regulation of genes (*Prdrx3*) to sensitize cells to apoptotic stimulus, accounts for male germ cell apoptosis as we observed previously [16,17].

Our earlier study revealed a significant increase of TUNEL-positive cells in stages VII-XIV with stages VII-VIII showing maximum effect following exogenous estradiol treatment [17]. Administration of 20 μg/kg/day of estradiol results in more than a 2-fold increase of rat intratesticular estrogen (135.2 ± 35.9 pg/gm) compared to that of the control (55.8 ± 7 pg/gm) [17], while administration of 100 μg/kg/day of estradiol leads to more than a 4-fold increase of rat intratesticular estrogen (246.7 ± 34 pg/gm) compared to that of the control [17]. Flow cytometry experiments in the present study indicate a significant decrease in the 2n cells (somatic and germ cells) and 4n cells (pachytene spermatocytes) by both 20 and 100 μg/kg/day of estradiol. This is consistent with our previous observations showing that estradiol induces cell apoptosis in the testis [16,17]. Notably we also found that estradiol caused a marked increase in the number of elongated spermatids, which is in agreement with our previous finding that spermiation failure occurred in rat testis exposed to estradiol [16,17]. In contrast, 20 and 100 μg/kg/day of estradiol have a different effect on the number of rat elongating and round spermatids. Physiological studies to demonstrate the involvement of estradiol in spermatogenesis and/or in its regulatory mechanisms need to be explored further.

## Conclusions

We have for the first time established a global gene database in the testes of rats following exposure to estradiol and found that numerous genes are involved in androgen and xenobiotic metabolism, maintenance of the cytoskeleton, intracellular transport, iron metabolism, endocytosis, and germ cell apoptosis. We have identified a list of genes that are regulated by estradiol and possess estrogen responsive elements. We also revealed that estradiol induced significant changes in testicular cell number. This study has thus dissected the molecular basis for spermiation failure and apoptosis seen after exogenous estradiol administration. The present study suggests involvement of estrogen in spermiation and phagocytic clearance of apoptotic germ cells. The study also highlights possible mechanisms through which adult exposure to environmental estrogen could affect fertility.

## List of abbreviations

(*Arc 1B*): actin related protein 2/3 complex, subunit 1B; (*Arpc5L*): actin related protein 2/3 complex, subunit 5-like (predicted); (*Ap2S1*): adaptor-related protein complex 2, sigma 1 subunit; (*Adh1*): alcohol dehydrogenase 1; (ANOVA): analysis of variance; (*Capg*): capping protein gelsolin like; (*Ces3*): carboxyesterase3; (*Csnk2b*): casein kinase 2 beta subunit; (*Cyp1b1*): cytochrome P450, family 1, subfamily b, polypeptide 1; (*Dncli2*): dynein cytoplasmic light intermediate polypeptide; (*Evl*): ENA Vasodilator Phosphoprotein; (ERα): estrogen receptors alpha; (ERβ): estrogen receptor beta; (EREs): estrogen responsive elements; (ESTs): expressed sequence tags; (*Hp*): haptoglobin; (*Hba-a1*): hemoglobin alpha adult chain 1; (*Hbb*): hemoglobin beta chain complex; (*Hsd11b1*): hydroxysteroid 11-beta dehydrogenase 1; (*Lamp 2*): lysosomal membrane glycoprotein 2; (*Nos3*): nitric oxide synthase 3, endothelial cell or eNOS; (*Prdx3 and Prdx6*): peroxiredoxin 3 and 6; (*PiCalm*): phosphatidylinositol binding clathrin assembly protein; (*Ralbp1*): Ral A binding protein; (*Rb1*): retinoblastoma; (*Star*): steroidogenic acute regulatory protein; *(Sult1a1)*: sulfotransferase family 1A, phenol-preferring, member 1; (*Stx5a*): syntaxin 5a; (*Stx8*): syntaxin 8; (*Top2a*): topoisomerase (DNA) 2 alpha; (*Trappc1*): trafficking protein particle complex 1 (predicted); (*Tgfbr3*): transforming growth factor, beta receptor 3; (*tubb5*): tubulin beta 5.

## Competing interests

The authors declare that they have no competing interests.

## Authors' contributions

NHB, ZH, and MD were responsible for designing and coordinating the study as well as for data interpretation and writing of the manuscript. ZH performed RNA isolation, microarray experiments, and data analysis. RDS, PN, SIT, and NKM performed real-time RT-PCR, in silico promoter and estrogen responsive elements analysis, and flow cytometry. ZH and RDS were involved in data collection and data analysis of the study. All authors read and approved the final manuscript.

## Supplementary Material

Additional file 1 **Supplemental Table 1**: Primer sequences used for real time RT-PCR. Click here for file

Additional file 2 **Supplemental Table 2**: Up-regulated genes and ESTs by estradiol. Click here for file

Additional file 3 **Supplemental Table 3**: Down-regulated genes and ESTs by estradiol. Click here for file

Additional file 4 **Supplemental Table 4**: The up-regulated genes by estradiol have an ERE sequence in the promoter region. Click here for file

Additional file 5 **Supplemental Table 5**: The down-regulated genes by estradiol have an ERE sequence in the promoter region. Click here for file
