# Supplementary material for: Effect of high intratesticular estrogen on global gene expression and testicular cell number in rats
Source: Reprod Biol Endocrinol. 2010 Jun 23;8:72. doi: 10.1186/1477-7827-8-72 (PMC2906496; doi:10.1186/1477-7827-8-72)
Supplement: Additional file 1 — Supplemental Table 1: Primer sequences used for real time RT-PCR. [file 1477-7827-8-72-S1.DOC]

| No | Name | Gene ID | Primers Sequences 5/ to 3/ |
| --- | --- | --- | --- |
| 1 | Carboxyesterase 3 | 113902 | Forward: tccaacgcttatgggctatc  Reverse: cttttggcagggtcatctgt |
| 2 | Topoisomerase Alpha | 360243 | Forward: TGCCCAGTTAGCTGGGTCAGTG  Reverse: TGAGCATTGTAAAGATGTACCT |
| 3 | Endothelial Nitric Oxide Synthase | 24600 | Forward: TACGAAGAATGGAAGTGGTTC  Reverse: TTGGCTCATCCATGTGGAACA |
| 4 | Ena Vasodialator phosphoprotein | 79115 | Forward: tcagggttgtaggggtcaag  Reverse: ttcatgatgttcagggcaaa |
| 5 | Retinoblastoma | 24708 | Forward: atgtgttccctctggaatgg  Reverse: gggagtgcctcttctttcaa |
| 6 | AP2 Sigma subunit 1 | 65046 | Forward: gagagacgagccagacgaag  Reverse: ctgtggcctagaccctgct |
| 7 | L19 |  | Forward: GAAATCGCCAATGCCAACTC Reverse: ACCTTCAGGTACAGGCTGTG. |

**Supplemental Table 1.** Primer sequences used for real time RT-PCR.
