# Supplementary material for: Effect of high intratesticular estrogen on global gene expression and testicular cell number in rats
Source: Reprod Biol Endocrinol. 2010 Jun 23;8:72. doi: 10.1186/1477-7827-8-72 (PMC2906496; doi:10.1186/1477-7827-8-72)
Supplement: Additional file 4 — Supplemental Table 4: The up-regulated genes by estradiol have an ERE sequence in the promoter region. [file 1477-7827-8-72-S4.DOC]

**Supplemental Table 4.** The up-regulated genes by estradiol have an ERE sequence in the promoter region.

| **Gene symbol** | **GeneID** | **Core sim.** | **Matrix sim.** | **Sequence** |  |
| --- | --- | --- | --- | --- | --- |
| Ace | 24310 | 1 | 0.929 | aggtgccAAGGacacaggg | |
| Add3 | 25230 | 1 | 0.841 | ttcgGTCAaaatacctctc | |
| Akap1 | 114124 | 1 | 0.939 | attcagcAAGGtcaggaac | |
| Akap1 | 114124 | 1 | 0.817 | accaGTCAgggtgtgctga | |
| Akap1 | 114124 | 1 | 0.843 | tgaaGTCAaagtgtgcaga | |
| Akap1 | 114124 | 1 | 0.876 | tccaggcAAGGccaagggg | |
| Akap1 | 114124 | 0.794 | 0.817 | cttgggcaccggGTCCtgg | |
| Akap12 | 83425 | 1 | 0.87 | tcaactgAAGGacacagaa | |
| Bbx_predicted | 303970 | 0.777 | 0.872 | tcccgccCAGGtcacagta | |
| Cct6a | 288620 | 1 | 0.818 | ttatGTCAtcctgcgctgt | |
| Cct6a | 288620 | 1 | 0.913 | cagagtcAAGGccaaggga | |
| Cpd | 25306 | 1 | 0.846 | agtgGTCAaactggccctc | |
| Cpd | 25306 | 1 | 0.862 | gagggccagtttGACCact | |
| Cpd | 25306 | 1 | 0.816 | acgggccaggtaGACCaac | |
| Crp | 25419 | 1 | 0.906 | aaagatcAAGGgcaagaag | |
| Dctn4 | 84428 | 1 | 0.881 | agagagcAAGGccagtgct | |
| Dhx36_predicted | 310461 | 1 | 0.941 | tgtttgcAAGGtcatggat | |
| Eef2 | 29565 | 1 | 0.909 | tgaggacAAGGacaaggag | |
| Eef2 | 29565 | 1 | 0.952 | gtgcgccAAGGtcaaatgc | |
| Eef2 | 29565 | 1 | 0.861 | gtcgGTCAacgtggacttg | |
| Hspca | 299331 | 0.794 | 0.853 | gctggtcaccttGTCCgcg | |
| Hspca | 299331 | 1 | 0.887 | cgcggacaaggtGACCagc | |
| Kras | 24525 | 1 | 0.819 | cccgggcaaaggGACCgtg | |
| Krt10 | 450225 | 1 | 0.845 | atagGTCAgcatattctat | |
| Krt10 | 450225 | 1 | 0.913 | tgctaccAAGGacagtgcc | |
| Mss4 | 304807 | 1 | 0.875 | aagtGTCAgcgtgacttgt | |
| Nedd4 | 25489 | 1 | 0.919 | cggcctcAAGGccagccac | |
| Nedd4 | 25489 | 1 | 0.919 | cggcctcAAGGccagccac | |
| Nol5 | 60373 | 1 | 0.987 | tgaactcAAGGtcagtctt | |
| Nol5 | 60373 | 1 | 0.88 | tcaataacattTGTTctct | |
| Nos3 | 24600 | 0.808 | 0.836 | cagggccaagccCACCcca | |
| Nup62 | 65274 | 1 | 0.831 | agaaGTCAtactgctctga | |
| Nup62 | 65274 | 1 | 0.835 | gtgggacaatgaGACCaag | |
| Picalm | 89816 | 1 | 0.889 | agtatgcAAGGccacgacc | |
| Plcl1 | 84587 | 0.808 | 0.838 | aagtgtcactatCACCcca | |
| Plcl1 | 84587 | 1 | 0.872 | gagcgagAAGGtcagatca | |
| Plcl1 | 84587 | 1 | 0.814 | gcgagacagcacGACCgcc | |
| Ppp2r2a | 117104 | 0.779 | 0.821 | cttgggcaccgcGGCCtgg | |
| Rb1 | 24708 | 0.767 | 0.81 | ctggGACAaggtgaactca | |
| Recc1 | 89809 | 1 | 0.933 | ccaaagcAAGGtcatatat | |
| Sfrs10 | 117259 | 1 | 0.975 | tgggttcAAGGtcaaccca | |
| Slc12a2 | 83629 | 1 | 0.94 | tatcaacAAGGtcaaacct | |
| Slc12a2 | 83629 | 1 | 0.837 | agcgggcaaggaGACCccc | |
| Slc12a2 | 83629 | 1 | 0.9 | cgtggccAAGGgcagcgag | |
| Socs6_predicted | 307200 | 1 | 0.852 | ttggGTCAcagtgcacctg | |
| Srpk2_predicted | 296753 | 1 | 0.984 | tgaactcAAGGtcagcctg | |
| Srpk2_predicted | 296753 | 0.794 | 0.867 | gtgagtcattttGTCCtta | |
| Srpk2_predicted | 296753 | 1 | 0.943 | ttataacAAGGtcagctac | |
| Srpk2_predicted | 296753 | 1 | 0.876 | gcctggcAAGGgcaaagcc | |
| Srpk2_predicted | 296753 | 1 | 0.851 | ttaaGTCAaggtgtgcaga | |
| Srpk2_predicted | 296753 | 1 | 0.844 | ggttGTCAtactgactttc | |
| Tcf12 | 25720 | 1 | 0.843 | acagGTCAtagtctactta | |
| Tgfbr3 | 29610 | 1 | 0.812 | ctgcgccatccgGACCccg | |
| Top2a | 360243 | 1 | 0.83 | caggGTCActatggcacac | |
| Top2a | 360243 | 1 | 0.844 | gtgtgccatagtGACCctg | |
| Tpo1 | 170907 | 1 | 0.841 | tccgggcagggcGACCgcg | |
| Tra1_predicted | 362862 | 1 | 0.873 | agcagatAAGGtcattgtc | |
| Tra1_predicted | 362862 | 1 | 0.839 | taagGTCAttgtcacgtcg | |
| Tra1_predicted | 362862 | 1 | 0.901 | tggccacAAGGgcacaggg | |
| Tra1_predicted | 362862 | 1 | 0.897 | gacgggcAAGGacatctct | |
| Tra1_predicted | 362862 | 1 | 0.877 | ctcaccgAAGGtcagcagg | |
| Tyro3 | 25232 | 0.779 | 0.822 | ccgggccaccccTACCtgc | |
| Tyro3 | 25232 | 1 | 0.818 | tcagGTCAccgtcctcccc | |
