# Supplementary material for: Effect of high intratesticular estrogen on global gene expression and testicular cell number in rats
Source: Reprod Biol Endocrinol. 2010 Jun 23;8:72. doi: 10.1186/1477-7827-8-72 (PMC2906496; doi:10.1186/1477-7827-8-72)
Supplement: Additional file 5 — Supplemental Table 5: The down-regulated genes by estradiol have an ERE sequence in the promoter region. [file 1477-7827-8-72-S5.DOC]

**Supplemental Table 5.** The down-regulated genes by estradiol have an ERE sequence in their promoter region

| **Gene symbol** | **GeneID** | **Core sim.** | **Matrix sim.** | **Sequence** |  |
| --- | --- | --- | --- | --- | --- |
| Acat1 | 25014 | 1 | 0.922 | gcttgtcAAGGgcagagag | |
| Acat2 | 308100 | 0.779 | 0.822 | cagggtcacatcGGCCtgc | |
| Acsl1 | 25288 | 1 | 0.913 | cattatcAAGGccagtggg | |
| Acsl1 | 25288 | 1 | 0.885 | gtaatcaAAGGtcagtcac | |
| Adh1 | 24172 | 0.808 | 0.831 | ctggggcagctcCACCttt | |
| Aes | 29466 | 1 | 0.996 | gaggttcAAGGtcacacag | |
| Aldoa | 24189 | 1 | 0.86 | tcagGTCAccaggccctag | |
| Aldoa | 24189 | 1 | 0.847 | ctagggcctggtGACCtga | |
| Aldoa | 24189 | 0.808 | 0.86 | tagggtcaccagAACCtcg | |
| Aldoa | 24189 | 0.779 | 0.843 | tatggccattttTACCtca |  |
| Aldoa | 24189 | 0.774 | 0.839 | caagGTGAgaatgatctgc | |
| Aldoa | 24189 | 1 | 0.863 | atggGTCAccttgcctaga | |
| Aldoa | 24189 | 1 | 0.844 | tctaggcaaggtGACCcat | |
| Aldoa | 24189 | 1 | 0.878 | tccgggcAAGGgcatcctg | |
| Aldoa | 24189 | 1 | 0.885 | ctcgacgAAGGtcactgta | |
| Anxa5 | 25673 | 1 | 0.813 | agaagacaccaaGACCtcc | |
| Anxa5 | 25673 | 1 | 0.874 | gggaagcAAGGccagccac | |
| Anxa5 | 25673 | 1 | 0.843 | caggGTCAgattggattgt | |
| Argbp2 | 114901 | 1 | 0.909 | ttatttcAAGGccattttt |  |
| Argbp2 | 114901 | 1 | 0.895 | aactaacAAGGacaaatga | |
| Arpc1b | 54227 | 1 | 0.878 | gtagGTCAtagtcccttgt |  |
| Capg | 297339 | 1 | 0.832 | agctGTCAcattgccctga | |
| Capg | 297339 | 1 | 0.836 | tcagGTCAtgcttagcttc |  |
| Crot | 83842 | 1 | 0.879 | ccaaaacAAGGgcagctct | |
| Ctss | 50654 | 1 | 0.87 | gaggagcAAGGgcaaatag | |
| Ctss | 50654 | 1 | 0.821 | tagagtcatttcGACCagc | |
| Ctss | 50654 | 1 | 0.88 | catgGTCAccgtgaagtgt | |
| Cyp1b1 | 25426 | 1 | 0.843 | ctggGTCAccctgagcaca | |
| Cyp1b1 | 25426 | 1 | 0.894 | gcagGTCAgtctgtccacg | |
| Cyp1b1 | 25426 | 1 | 0.895 | cgtggacagactGACCtgc | |
| Cyp1b1 | 25426 | 1 | 0.902 | gttcttgAAGGtcaggggt | |
| Dlg7_predicted | 289997 | 1 | 0.872 | cgagGTCAgcctgggctaa | |
| Dlg7_predicted | 289997 | 0.761 | 0.878 | tgaactcGAGGtcagcctg | |
| Dlg7_predicted | 289997 | 1 | 0.904 | gacgtttAAGGtcattctt |  |
| Dpep1 | 94199 | 1 | 0.903 | aaatcccAAGGccacctca | |
| Dpep1 | 94199 | 1 | 0.836 | aaagGTCAcagtcggctat | |
| Dpep1 | 94199 | 1 | 0.874 | gagaagaAAGGtcacagtc | |
| Dpep1 | 94199 | 1 | 0.881 | cagggtcaggagGACCctt | |
| Dpep1 | 94199 | 1 | 0.853 | aagggtcctcctGACCctg | |
| Dpep1 | 94199 | 1 | 0.911 | acagctcAAGGccaacatg | |
| Ercc1_predicted | 292673 | 0.79 | 0.839 | caagGGCAgcctgatctac | |
| Ercc1_predicted | 292673 | 1 | 0.924 | tgagctcAAGGgcagcctg | |
| Evl | 79115 | 1 | 0.871 | tggaacaAAGGtcaaaggc | |
| F2r | 25439 | 1 | 0.885 | ggagcgaAAGGtcactcca | |
| Fabp3 | 79131 | 1 | 0.933 | gggaagcAAGGtcatgttc | |
| Fabp3 | 79131 | 1 | 0.872 | ctgggagaacatGACCttg | |
| Fabp3 | 79131 | 1 | 0.839 | cgcgGTCAccataagtaga | |
| Fmod | 64507 | 1 | 0.818 | attcggcagcaaGACCtca | |
| Fmod | 64507 | 1 | 0.851 | cgtgGTCActctgaacggt | |
| Gad1 | 24379 | 0.794 | 0.823 | cagggacacctgGTCCcca | |
| Gad1 | 24379 | 1 | 0.88 | tgagagcAAGGgcagataa | |
| Gnai2 | 81664 | 0.779 | 0.838 | aagggtcaggaaTACCtgc | |
| Gnb1 | 24400 | 1 | 0.831 | caaaGTCAgcctggtctac | |
| Gnb1 | 24400 | 1 | 0.813 | atgagtgaacttGACCagc | |
| Got1 | 24401 | 1 | 0.842 | gcgggtgaccttGACCatc | |
| Got1 | 24401 | 1 | 0.993 | gatggtcAAGGtcacccgc | |
| Got1 | 24401 | 1 | 0.815 | ctgggtcctggaGACCtgg | |
| Got1 | 24401 | 1 | 0.874 | gacccagAAGGtcaggagg | |
| Got1 | 24401 | 1 | 0.834 | caatGTCAgaatgccgtgt | |
| Gucy1b3 | 25202 | 1 | 0.965 | cacagacAAGGtcacacac | |
| Hbb | 24440 | 0.808 | 0.847 | tggggtcaacaaAACCtcc | |
| hepatocyte | --- | 1 | 0.891 | aagtccaAAGGtcacctaa | |
| Hp | 24464 | 1 | 0.887 | ccttgctAAGGtcagtgac | |
| Hp | 24464 | 1 | 0.839 | tcggGTCAtggtgctccct | |
| Hp | 24464 | 1 | 0.826 | agggagcaccatGACCcga | |
| Hp | 24464 | 1 | 0.887 | ccttgctAAGGtcagtgac | |
| Hsd11b1 | 25116 | 1 | 0.875 | ttcttgcAAGGccattgct |  |
| Hsd11b1 | 25116 | 1 | 0.893 | cagagacAAGGccagagag | |
| Hspd1 | 63868 | 0.774 | 0.869 | tttgGTGAcgatgacctct |  |
| Hspd1 | 63868 | 0.808 | 0.882 | agaggtcatcgtCACCaaa | |
| Hspd1 | 63868 | 0.808 | 0.847 | cttggtcagctaAACCtaa | |
| Hspd1 | 63868 | 1 | 0.837 | cttgGTCAtaattgctgaa |  |
| Igfbp2 | 25662 | 1 | 0.98 | ctcactcAAGGtcattgtt |  |
| Igfbp2 | 25662 | 1 | 0.874 | aatttctAAGGtcaagatt |  |
| Igfbp2 | 25662 | 1 | 0.857 | taagGTCAagattgctttt |  |
| Ins2 | 24506 | 1 | 0.912 | ctcagccAAGGacaaagaa | |
| Ins2 | 24506 | 0.777 | 0.876 | tatcttcCAGGtcattgtt |  |
| Lgals1 | 56646 | 1 | 0.848 | gttgGTCAgggtatcccag | |
| Lgals1 | 56646 | 1 | 0.825 | ctgggataccctGACCaac | |
| Lgals1 | 56646 | 0.808 | 0.836 | tggggtcatttcCACCctg |  |
| Lgals3 | 83781 | 0.794 | 0.811 | cagggacacctgGTCCtcc | |
| Map2k5 | 29568 | 1 | 0.881 | cttgGTCActgttatctgt |  |
| Map2k5 | 29568 | 0.808 | 0.874 | gtgggacattctCACCtgc | |
| Nenf | 289380 | 1 | 0.833 | tgagggcacccgGACCagc | |
| Nkiras2_predicted | 287707 | 1 | 0.938 | tgatttcAAGGacagccaa | |
| Nkiras2_predicted | 287707 | 1 | 0.938 | tgatttcAAGGacagccaa | |
| Nrd1 | 25499 | 1 | 0.92 | tgagttcAAGGccagcctg | |
| Nrd1 | 25499 | 0.779 | 0.835 | caaggccagcctGGCCtac | |
| Nrd1 | 25499 | 0.779 | 0.849 | gtaggccaggctGGCCttg | |
| Oxr1 | 117520 | 1 | 0.944 | catcttcAAGGacacttat | |
| Oxr1 | 117520 | 1 | 0.859 | ccagGTCAcattaagcttc | |
| Pbef1 | 297508 | 0.777 | 0.872 | aggcgccCAGGtcacgcgc | |
| Pc | 25104 | 1 | 0.863 | aaggGTCAcattaccctga | |
| Pc | 25104 | 1 | 0.895 | gaatcacAAGGccaggcat | |
| Pcm1 | 81740 | 1 | 0.891 | atagGTCAgattaactttt |  |
| Pgk1 | 24644 | 1 | 0.888 | tgtgtgcAAGGccagaggc | |
| Plcb1 | 24654 | 1 | 0.951 | ttagggcAAGGtcagaact | |
| Plcb1 | 24654 | 0.808 | 0.819 | aatggacacaatAACCgtg | |
| Plcb1 | 24654 | 1 | 0.863 | agagGTCAtgattactcgg | |
| Plcb1 | 24654 | 1 | 0.83 | ccgagtaatcatGACCtct | |
| Plcb1 | 24654 | 1 | 0.824 | cagagagaaactGACCtga | |
| Prdx3 | 64371 | 1 | 0.9 | gctgGTCAgagtgtctgtt |  |
| Prdx3 | 64371 | 1 | 0.825 | aacagacactctGACCagc | |
| Psat1 | 293820 | 1 | 0.863 | ccggGTCAcaatcctctga | |
| Psme4 | 498433 | 1 | 0.862 | ttagGTCAtaatcttttga |  |
| Psme4 | 498433 | 1 | 0.916 | ggaattcAAGGccagtttg | |
| Ptprn2 | 29714 | 1 | 0.869 | aggagccagggtGACCgcg | |
| Ptprn2 | 29714 | 1 | 0.845 | cagtGTCAagatgagtttg | |
| Ptprn2 | 29714 | 1 | 0.858 | ggacGTCAgagtggcttga | |
| Ptprn2 | 29714 | 0.794 | 0.847 | gatgggcagcatGTCCtgt | |
| Ptprn2 | 29714 | 1 | 0.854 | tctgGTCAacctgtctatc |  |
| Ptprn2 | 29714 | 1 | 0.862 | gatagacaggttGACCaga | |
| Ptprn2 | 29714 | 1 | 0.877 | agcctcaAAGGtcattggt | |
| Ptprn2 | 29714 | 1 | 0.905 | taccaacAAGGacagtggc | |
| Ralbp1 | 84014 | 1 | 0.891 | gaggtccAAGGgcatccat | |
| Ralbp1 | 84014 | 1 | 0.883 | tcttcagAAGGtcactgtg | |
| Ralbp1 | 84014 | 1 | 0.872 | agcagtaAAGGacacgaaa | |
| RGD1562406_predicted | 288559 | 1 | 0.844 | tgtggccaccggGACCctg | |
| RGD1562406_predicted | 288559 | 0.808 | 0.812 | atgggaaaacttAACCtgg | |
| RGD1562406_predicted | 288559 | 1 | 0.86 | catgGTCAccaagtccttg | |
| RGD1562406_predicted | 288559 | 1 | 0.93 | ggagttcAAGGccactgtt | |
| RGD1562406_predicted | 288559 | 1 | 0.916 | ggggttcAAGGccattcct | |
| Rpl39 | 25347 | 1 | 0.871 | cttctgaAAGGtcacctac | |
| Rpn2 | 64701 | 1 | 0.891 | cccggcaAAGGtcagtctc | |
| Rpn2 | 64701 | 1 | 0.874 | gaagtgcAAGGccatcatt | |
| Rpn2 | 64701 | 0.75 | 0.801 | tgggaatggtgTGGTcttg | |
| Rpn2 | 64701 | 1 | 0.862 | gaggggaatggtGACCcca | |
| Sepp1 | 29360 | 1 | 0.891 | gagagccAAGGccaaagcc | |
| Sfxn3 | 65042 | 0.808 | 0.819 | gtgggtcaggcgAACCagt | |
| Sfxn3 | 65042 | 1 | 0.82 | tgggGTCAccttgagcatc | |
| Sfxn3 | 65042 | 1 | 0.818 | gatgctcaaggtGACCcca | |
| Sfxn3 | 65042 | 1 | 0.876 | cttctgcAAGGgcagcaag | |
| Sh3kbp1 | 84357 | 0.779 | 0.864 | gaaagtcaccatGGCCtga | |
| Sh3kbp1 | 84357 | 0.808 | 0.825 | gcggggcatgacCACCcag | |
| Sh3kbp1 | 84357 | 1 | 0.901 | cagccccAAGGccagagaa | |
| Sh3kbp1 | 84357 | 1 | 0.884 | cttgtcaAAGGtcagctac | |
| Sh3kbp1 | 84357 | 1 | 0.84 | gctgGTCAatttggccatt |  |
| Sh3kbp1 | 84357 | 1 | 0.845 | aatggccaaattGACCagc | |
| Sh3kbp1 | 84357 | 1 | 0.929 | ggccctcAAGGccactgcc | |
| Slc3a2 | 50567 | 0.808 | 0.832 | ctgggtcacaacCACCaaa | |
| Snx4_predicted | 360725 | 1 | 0.87 | agaagcgAAGGtcaagtga | |
| Sos1 | 313845 | 1 | 0.959 | atcatccAAGGtcagcctc | |
| Sos1 | 313845 | 1 | 0.812 | caagGTCAgcctcagccat | |
| Sos1 | 313845 | 0.808 | 0.893 | caggggcaagttCACCcta | |
| Sos1 | 313845 | 1 | 0.943 | gaagttcAAGGacaggaat | |
| Star | 25557 | 1 | 0.889 | aggaaccAAGGccagccag | |
| Star | 25557 | 1 | 0.92 | tgagttcAAGGccagcctg | |
| Stk38 | 361813 | 0.779 | 0.811 | agaggacagccaGGCCtta | |
| Stk38 | 361813 | 1 | 0.82 | tagaGTCAcagtggactgt | |
| Stk38 | 361813 | 1 | 0.858 | tttgGTCAttgtcaccctc |  |
| Stk38 | 361813 | 1 | 0.846 | gagggtgacaatGACCaaa | |
| Stx5a | 65134 | 1 | 0.909 | cgagcccAAGGccacccgc | |
| Sult1a1 | 83783 | 1 | 0.947 | caggagcAAGGtcagggaa | |
| Sult1a1 | 83783 | 0.767 | 0.85 | acagGACAaggtggccacc | |
| Sult1a1 | 83783 | 0.794 | 0.837 | ggtggccaccttGTCCtgt | |
| Tars | 294810 | 1 | 0.884 | tggacacAAGGccaatgaa | |
| Tars | 294810 | 1 | 0.813 | agagGTCAtccagagccgg | |
| Tars | 294810 | 1 | 0.977 | tgagttcAAGGtcatcctt |  |
| Tars | 294810 | 1 | 0.887 | gagagaaAAGGtcactgga | |
| Thop1 | 64517 | 1 | 0.965 | tcctgccAAGGtcacctgc | |
| Thop1 | 64517 | 1 | 0.909 | caaactgAAGGtcaccctc | |
| Thop1 | 64517 | 1 | 0.981 | agagctcAAGGtcatcctt | |
| Thop1 | 64517 | 0.806 | 0.856 | tgagGTAAcgatgtcatga | |
| Thop1 | 64517 | 0.779 | 0.815 | tcatgacatcgtTACCtca | |
| Txnrd1 | 58819 | 1 | 0.84 | tgggGTCActatgcagtca | |
| Txnrd1 | 58819 | 1 | 0.865 | cctgGTCAtcatgccagtt | |
| Txnrd1 | 58819 | 1 | 0.835 | aactggcatgatGACCagg | |
| Txnrd1 | 58819 | 1 | 0.902 | ggagctaAAGGtcatcacc | |
| Unr | 117180 | 1 | 0.934 | cacaatcAAGGacagaaat | |
| Unr | 117180 | 0.808 | 0.831 | aatagtcagtttCACCcca | |
| Wdfy3_predicted | 305164 | 0.777 | 0.885 | tcaagtcCAGGtcaggtgc | |
| Wdfy3_predicted | 305164 | 1 | 0.893 | cctaatgAAGGtcagttgg | |
| Wdfy3_predicted | 305164 | 0.761 | 0.883 | ggtgttcTAGGtcagggtt |  |
| Wdfy3_predicted | 305164 | 1 | 0.833 | ctagGTCAgggttcacttc | |
| Wdfy3_predicted | 305164 | 0.794 | 0.841 | gagagtcagcatGTCCccc | |
| Wdfy3_predicted | 305164 | 0.808 | 0.81 | aagagacatcgaAACCtta | |
| Wiz_predicted | 314598 | 1 | 0.87 | gagaaaaAAGGtcagttat | |
| Wiz_predicted | 314598 | 0.794 | 0.825 | atgagtcaggctGTCCacc | |
